# Creativity and Cognitive Skills among *Millennials*: Thinking Too Much and Creating Too Little

**DOI:** 10.3389/fpsyg.2016.01626

**Published:** 2016-10-25

**Authors:** Brice Corgnet, Antonio M. Espín, Roberto Hernán-González

**Affiliations:** ^1^EMLYON Business School, Univ Lyon, GATE L-SE UMR 5824Ecully, France; ^2^Economics Department, Middlesex University Business SchoolLondon, UK; ^3^Granada Lab of Behavioral Economics, Universidad de GranadaGranada, Spain; ^4^Business School, University of NottinghamNottingham, UK

**Keywords:** creativity, cognitive reflection, intelligence, cognition, intuition

## Abstract

Organizations crucially need the creative talent of millennials but are reluctant to hire them because of their supposed lack of diligence. Recent studies have shown that hiring diligent millennials requires selecting those who score high on the Cognitive Reflection Test (CRT) and thus rely on effortful thinking rather than intuition. A central question is to assess whether the push for recruiting diligent millennials using criteria such as cognitive reflection can ultimately hamper the recruitment of creative workers. To answer this question, we study the relationship between millennials' creativity and their performance on fluid intelligence (Raven) and cognitive reflection (CRT) tests. The good news for recruiters is that we report, in line with previous research, evidence of a positive relationship of fluid intelligence, and to a lesser extent cognitive reflection, with *convergent creative thinking*. In addition, we observe a positive effect of fluid intelligence on originality and elaboration measures of *divergent creative thinking*. The bad news for recruiters is the inverted U-shape relationship between cognitive reflection and fluency and flexibility measures of *divergent creative thinking*. This suggests that thinking too much may hinder important dimensions of creative thinking. Diligent and creative workers may thus be a rare find.

## Introduction

Evidence from a recent survey reports that managers are three times more likely to hire a mature worker than to hire a *millennial* (born between 1980 and 2000; Rainer and Rainer, 2011) despite desperately needing their creative talent^1^. Mature workers are appealing to recruiters because they are seen as more reliable and more committed than millennials. The dilemma for managers is thus to hire millennials that are both diligent and creative.

Recent studies have shown that firms can secure the hiring of diligent millennials by relying on measures of cognitive skills. For example, intelligence has been found to be the main predictor of overall work performance in a wide variety of occupations and across age and gender (e.g., Hunter and Hunter, 1984; Olea and Ree, 1994; see Schmidt, 2009 for a review). Standard measures of cognitive ability have been found to correlate positively with task performance (Schmidt et al., 1986; Murphy, 1989) and negatively with counterproductive work behaviors such as theft or absenteeism (Dilchert et al., 2007). Moreover, the results of a recent study suggest that these effects may be mediated by individuals' cognitive styles (Corgnet et al., 2015b). In particular, Corgnet et al. (2015b) find that millennials characterized by a more reflective style (as measured by the Cognitive Reflection Test; Frederick, 2005) are more diligent, displaying higher levels of task performance and lower levels of counterproductive work behaviors^2^. A crucial caveat is whether hiring millennials based on cognitive measures may ultimately select less creative workers. To address this point we need to assess the relationship between cognitive skills and creativity.

Traditionally, intelligence, and creativity have been considered to be unrelated (Getzels and Jackson, 1962; Wallach and Kogan, 1965; Batey and Furnham, 2006; Sawyer, 2006; Weisberg, 2006; Runco, 2007; Kaufman, 2009; Kim et al., 2010). In a meta-analysis, Kim (2005) finds that the correlation between creativity test scores and IQ varies widely and is, on average, small (*r* = 0.174).

However, a growing consensus has emerged in recent research stressing a close relationship between intelligence and creative performance (see Silvia, 2015, for a review). This emerging consensus heavily relies on recent studies that have employed more sophisticated statistical techniques and more robust assessment methods than prior research on the topic. For example, the use of latent variable models has allowed researchers to uncover a positive and significant relationship between creativity and intelligence using data from previous studies that reported non-significant correlations (Silvia, 2008b). The recent wave of research on intelligence and creativity has also improved upon traditional assessment of creativity that exclusively relied on scoring methods based on the originality and uniqueness of responses in creative tasks (such as finding unusual uses for an object). These traditional scoring methods are imprecise because they confound several factors, such as fluency and sample size (Hocevar, 1979; Silvia et al., 2008), and can thus lead to inaccurate estimates of the relationship between intelligence and creativity (Silvia, 2008a; Nusbaum and Silvia, 2011). The results of this new wave of research on creativity and intelligence have been taken as evidence that executive cognition is undoubtedly beneficial to creative thinking (Silvia, 2015).

Yet, although there is an obvious link between intelligence and executive cognition, from the point of view of modern dual-process theory (Evans, 2008, 2009; Stanovich, 2009, 2010; Evans and Stanovich, 2013), one should distinguish between algorithmic and reflective cognitive processes. Algorithmic processes are typically associated with computational efficiency and are measured by standard intelligence tests whereas reflective processing is associated with a disposition to employ the resources of the algorithmic mind, that is, to switch from autonomous “Type 1” thought to analytic “Type 2” (working memory-dependent) thought. The reflective mind thus has a disposition-based definition (“cognitive styles”, reflective vs. intuitive) and is not adequately measured by standard intelligence tests (which assess “cognitive ability”) but by tasks of cognitive reflection like the Cognitive Reflection Test (CRT; Frederick, 2005). Individuals characterized by a more reflective mind tend to show higher levels of self-control and lower levels of “cognitive impulsivity” (Frederick, 2005; Kahneman and Frederick, 2007; Cokely and Kelley, 2009; Oechssler et al., 2009; Toplak et al., 2011; Brañas-Garza et al., 2012).

From this perspective, one can conjecture that cognitive reflection may relate negatively to creativity. This is the case because a number of studies suggest that the capacity to control one's attention and behavior may even be detrimental for creative thinking (for a review, see Wiley and Jarosz, 2012a). For example, creative problem solving has been shown to relate positively to moderate alcohol intoxication (Jarosz et al., 2012), which is known to impair inhibition and attentional control (Peterson et al., 1990; Kovacevic et al., 2012; Marinkovic et al., 2012). Similarly, an “experiential” thinking style (which maps onto Type 1 processing) has been found to correlate positively with creative performance (Norris and Epstein, 2011).

As mentioned, past literature arrived at conflicting conclusions regarding whether executive cognition favors (e.g., Nusbaum and Silvia, 2011; Beaty and Silvia, 2012; Silvia, 2015) or hampers (e.g., Eysenck, 1993; Kim et al., 2007; Ricks et al., 2007; Norris and Epstein, 2011; Jarosz et al., 2012; Wiley and Jarosz, 2012b) creative thinking. Dual-process theory can reconcile these apparently conflicting findings by positing that creativity may be generated by a mix of Type 1 and Type 2 processes (Allen and Thomas, 2011; Ball et al., 2015; Barr et al., 2015; see Sowden et al., 2015, for a review). It follows that the dual-process approach lays out a promising research agenda based on assessing the exact mix of Type 1 and Type 2 processes that bolsters creativity as well as analyzing separately the effect of algorithmic and reflective Type 2 processes on creative thinking.

Following a dual-process approach, Barr et al. (2015) find experimental evidence of an important effect of controlled Type 2 analytic processes on both *convergent* and *divergent* (Guilford, 1967) creative thinking. In particular, they find that both cognitive ability (measured as the combination of numeracy and verbal skills) and reflective cognitive style (average of scores in the CRT and base-rate problem tasks) covary positively with one's capacity to make remote associations, that is, with convergent creative thinking. Regarding divergent creative thinking, Barr et al. (2015) show that cognitive ability but *not* cognitive reflection predicts higher originality scores in an alternate uses task. Fluency in the latter task, however, was not correlated with either cognitive measure.

In this paper, we use a similar approach to Barr et al. (2015) and investigate how both types of cognitive processes affect creativity. In particular, we analyze how cognitive abilities (measured using Raven as a test of fluid intelligence) and cognitive styles (intuitive vs. reflective; as measured by the CRT) relate to convergent and divergent creative thinking. We extend Barr et al. (2015) by analyzing other measures of divergent thinking such as flexibility and elaboration and by exploring possible non-linearities between creativity and cognitive measures.

Given the conflicting results regarding whether executive cognition is beneficial or detrimental for creative thinking, we conjecture that there might exist a non-linear relationship between different measures of creativity and cognition. Specifically, it might be that a minimum level of executive cognition is necessary for creative performance but, beyond some level, the relationship disappears or even turns negative. This might explain why previous findings seem to be inconsistent. A related line of reasoning has been proposed in the so-called “threshold hypothesis” of the relationship between IQ and creativity (Guilford, 1967; Jauk et al., 2013). The threshold hypothesis states that intelligence is positively related to creative thinking for low IQ levels but the relationship blurs for high IQ levels. Similar arguments arise in recent accounts of the “mad genius hypothesis”: moderate levels of inhibitory or top-down control dysfunction, characteristic of subclinical psychiatric populations (e.g., mild ADHD and schizophrenia disorders), can spur creativity under some conditions whereas clinical-severe levels typically lead to impoverished creative thinking (Schuldberg, 2005; Abraham et al., 2007; Jaracz et al., 2012; Acar and Sen, 2013; Abraham, 2014).

## Methods

### Participants and general protocol

Participants were 150 students (46.67% female; age: mean ± *SD* = 20.23 ± 1.96) from Chapman University in the U.S. These participants were recruited from a database of more than 2000 students. Invitations to participate in the current study were sent to a random subset of the whole database. This study is part of a larger research program on cognition and economic decision making. The local Institutional Review Board approved of this research. All participants provided written informed consent prior to participating. We conducted a total of 12 sessions, nine had 12 participants and three had 14 participants. On average, sessions lasted for 45 min. All subjects completed the same tasks in the following order: (1) CRT, (2) Raven test, (3) Remote associates task, (4) Alternate uses task. Subjects had 6 min to complete each task and a 2-min break after completing the Raven test.

### Measures

#### Cognitive ability assessment

Participants completed a subset of Raven progressive matrices test (Raven, 1936). Specifically, we used the odd number of the last three series of matrices (Jaeggi et al., 2010; Corgnet et al., 2015a). The number of matrices correctly solved in the Raven test (in our sample, ranging from 9 to 18, mean ± *SD* = 14.40 ± 2.42 for males and 14.47 ± 2.16 for females) is a conventional measure of cognitive ability. This test captures an important aspect of cognitive processing which is referred to as fluid intelligence and is closely related to algorithmic thinking (Stanovich, 2009, 2010).

#### Cognitive style assessment

We measured the participants' tendency to rely on intuition vs. reflection using the CRT introduced by Frederick (2005). The test is characterized by the existence of an incorrect response which automatically comes to mind but has to be overridden in order to find the correct solution. To the original CRT questions, we added four questions recently developed by Toplak et al. (2014). This extended task (see Text S1) will allow us to uncover potentially non-linear relationships that would be hard to observe using the classical three-item task (Frederick, 2005). In Table S1, we display the proportion of subjects answering each question correctly, split by gender. As expected, males performed better in the test than females (Frederick, 2005; Bosch-Domènech et al., 2014). Our measure of cognitive reflection is given by the total number of correct answers (from 0 to 7). The full distribution of correct answers by males (mean ± *SD* = 4.09 ± 2.31) and females (mean ± *SD* = 2.89 ± 2.03) is provided in Figure S1.

#### Convergent creative thinking

We used a subset of the Remote Associate Test (RAT; Mednick, 1962) to measure subjects' ability to make remote associations. In particular, subjects were shown 13 sets of three words (e.g., widow-bite-monkey) and asked to find a word which relates to all the three words provided (in this example the solution is “spider”). Our measure of convergent thinking is the number of problems correctly solved (from 0 to 13).

#### Divergent creative thinking

We measured divergent thinking using a variant of the Alternate Uses Task (AUT; Guilford, 1967). Participants were instructed to provide as many unusual uses of a pen as possible during 6 min. We construct four different measures of divergent thinking: *fluency, originality, flexibility,* and *elaboration*. We measured *fluency* as the total number of answers provided by a participant. Three raters were presented with a random list of answers and asked to score the degree of originality of each entry using a 1 (*not at all*) to 5 (*very much*) Likert scale. We computed *originality* as the sum of the average score of the three raters for all the entries provided by a participant, divided by the total number of answers. Following Troyer and Moscovitch (2006) and Gilhooly et al. (2007), all the answers were classified in broad differentiated categories (e.g., uses of the pen as cloth or hair accessories). Then, *flexibility* was measured as the number of different categories provided by each participant. Finally, *elaboration* refers to the average amount of detail (from 0 to 2) provided by each participant.

### Statistical analysis

For the data analysis, we start by showing the descriptive statistics of all the measures used and their zero-order correlations. To further assess the relationships between creativity and cognitive measures, we first provide a graphical representation using LOWESS smoothing (Cleveland, 1979; Cleveland and McGill, 1985). We then run ordinary least squares regressions which allow us to test the statistical significance of the linear and non-linear relationships which were shown in the LOWESS graphs. All the analyses were performed using Stata 14.0.

## Results

### Descriptive statistics and correlations

Means, standard deviations, and correlations are shown in Table 1. Unsurprisingly, we find moderate positive correlation between the number of correct answers in the CRT and Raven tests (*r* = 0.26, *p* < 0.01) which suggests that CRT and Raven are not entirely measuring the same cognitive skills (Frederick, 2005; Stanovich, 2009, 2010). Similarly, the different measures of divergent thinking (AUT) are significantly correlated (all *p*'s < 0.01), except for *originality* and *flexibility* (*p* = 0.28).

**Table 1:** **Descriptive statistics and Pearson correlations**.

	**Mean**	**Std Dev**	**[1]**	**[2]**	**[3]**	**[4a]**	**[4b]**	**[4c]**	**[4d]**
**COGNITIVE MEASURES**									
1. Raven	14.43	2.30	–						
2. CRT	3.53	2.26	0.26^**^	–					
**CREATIVITY**									
3. RAT	3.69	2.97	0.23^**^	0.17^*^	–				
4. AUT									
4.a. Originality	1.33	0.54	0.20^*^	0.09	0.14	–			
4.b. Fluency	16.47	8.90	−0.17^*^	−0.06	−0.06	−0.25^**^	–		
4.c. Flexibility	11.17	4.22	−0.10	−0.01	−0.02	−0.09	0.85^***^	−	
4.d. Elaboration	0.23	0.29	0.26^**^	0.06	0.10	0.37^***^	−0.36^***^	−0.31^***^	−

*N = 150*,

* *p < 0.05*,

** *p < 0.01*,

*** *p < 0.001*.

Regarding our cognitive measures, we find that both Raven (*p* < 0.01) and CRT scores (*p* = 0.03) are positively correlated with convergent thinking (RAT). However, the relationship between cognitive skills and divergent thinking is more complicated. High levels of cognitive ability (Raven) relate positively with *originality* (*p* = 0.01) and *elaboration* (*p* < 0.01), but negatively with the number of answers provided (*fluency*; *p* = 0.04) and non-correlated with *flexibility* (*p* = 0.20). Finally, we do not find a significant correlation between cognitive styles (CRT scores) and any measure of divergent thinking (all *p'*s > 0.26).

### Non-linear effects and regression analysis

**Figure 1:**
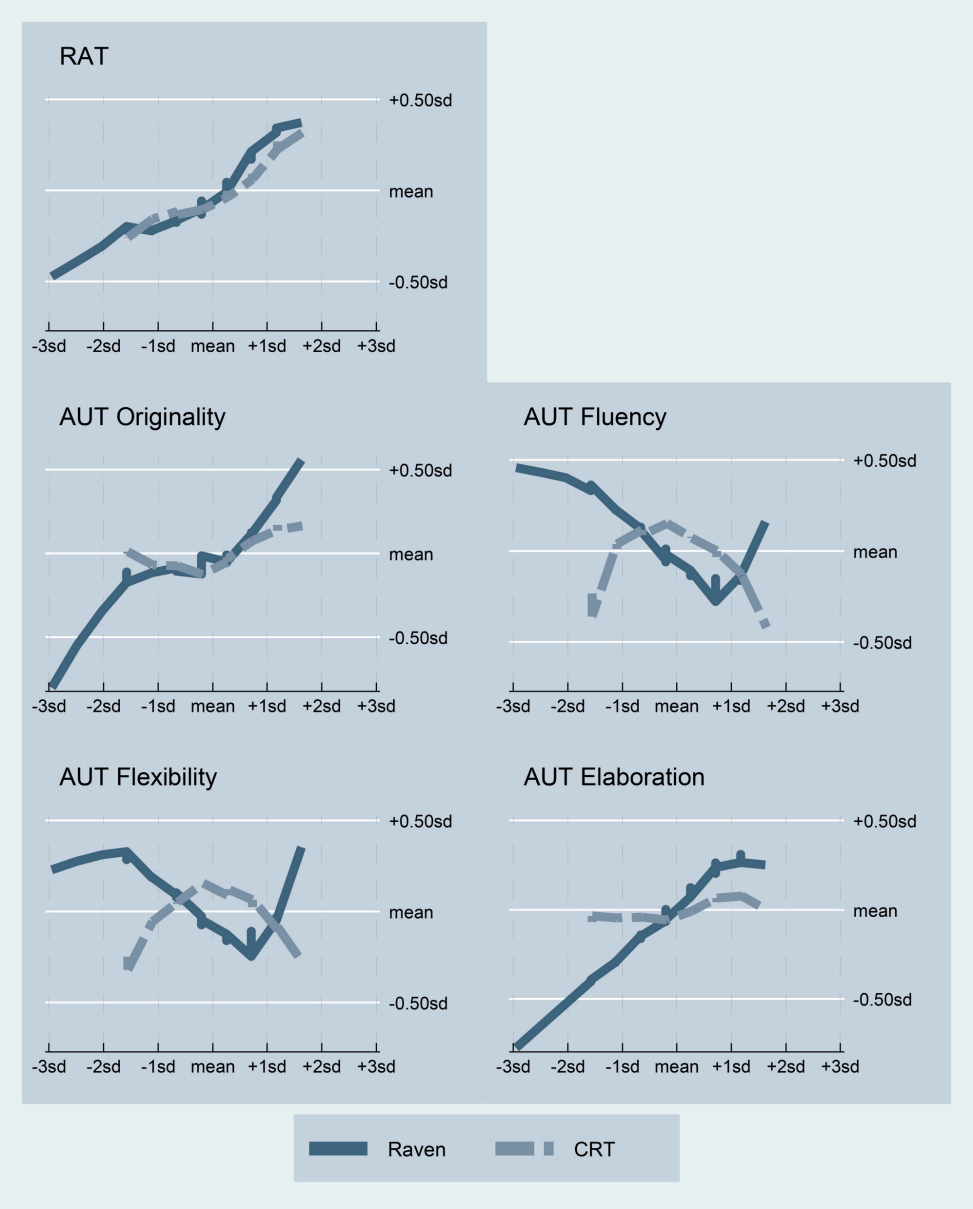
**Relationship between cognitive measures and creative thinking**. The relationships are represented using locally weighted smoothing (LOWESS) techniques. All variables are standardized.

We now turn to the study of possible non-linear relationships between our measures of cognition and creativity. Figure 1 displays all the relationships under study using LOWESS (bandwidth = 0.8; Cleveland, 1979; Cleveland and McGill, 1985). LOWESS is a model-free smoothing technique based on locally-weighted regressions which can detect both linear and non-linear relationships. In order to compare the effect sizes, we standardize all measures (standard deviations from the mean). We also ran ordinary least squares regressions to assess the statistical significance of the observed relationships. In Tables S2–S6, we present the results of a series of regressions in which we estimated both linear and quadratic effects of each of the predictors (Raven and CRT) separately on each creativity measure (columns [1] to [4]). From these regressions, we selected the models with the best fit, either linear or quadratic in each case, using the Akaike Information Criterion (AIC) and report them in summary Table 2. In addition, we ran similar regressions in which both predictors (linear and quadratic terms) are included simultaneously (columns [5] and [6] in Tables S2–S6) in order to test for possible mediation or confounding effects. The interaction between CRT and Raven scores is never significant in predicting creativity (all *p*'s > 0.3) and is thus not reported in the tables for the sake of brevity. The results remain qualitatively similar if we also control for gender and age.

**Table 2:** **The effect of cognitive abilities and cognitive styles on creativity (best fitting models)**.

	**RAT**		**AUT Originality**		**AUT Fluency**		**AUT Flexibility**		**AUT Elaboration**	
	**[Raven]**	**[CRT]**	**[Raven]**	**[CRT]**	**[Raven]**	**[CRT]**	**[Raven]**	**[CRT]**	**[Raven]**	**[CRT]**
Raven_std_	0.219^***^		0.195^***^		−0.162^**^		−0.099		0.246^***^	
	(0.076)		(0.071)		(0.074)		(0.081)		(0.067)	
Raven^2^_std_										
										
CRT_std_		0.169^**^		0.089		−0.043		0.011		0.053
		(0.076)		(0.082)		(0.066)		(0.073)		(0.082)
CRT^2^_std_						−0.207^***^		−0.194^**^		
						(0.076)		(0.079)		
Constant	0.001	−0.010	0.001	−0.005	−0.001	0.224	−0.001	0.206	0.001	−0.003
	(0.080)	(0.080)	(0.080)	(0.081)	(0.081)	(0.136)	(0.082)	(0.127)	(0.079)	(0.082)
*F*	8.229	4.891	7.562	1.179	4.732	4.255	1.490	2.990	13.302	0.420
prob > *F*	0.005	0.029	0.007	0.279	0.031	0.016	0.224	0.053	0.000	0.518
*R*^2^	0.053	0.030	0.042	0.008	0.029	0.039	0.011	0.031	0.067	0.003
Ll	−208.277	−210.018	−209.129	−211.706	−210.139	−209.359	−211.522	−210.000	−207.174	−212.112
AIC	420.555	424.036	422.258	427.411	424.278	424.717	427.044	426.000	418.348	428.225

OLS estimates. N = 150. All variables are standardized. Robust standard errors are shown in parentheses. See Tables S2–S6 for alternative specifications. *p < 0.05,

** *p < 0.01*,

*** p < 0.001.

The models with the best fit (Table 2) report a positive linear relationship of convergent thinking (RAT) with both Raven (*p* < 0.01) and CRT scores (*p* = 0.03), which is consistent with the positive and significant correlations reported in the previous section. Effect sizes are substantial: in both cases, one *SD* increase in the predictor is associated with about 20% of one *SD* increase in RAT (0.22 and 0.17 for Raven and CRT, respectively; see coefficients in Table 2). Interestingly, the effect of Raven on RAT remains significant (*p* = 0.02) if we include both Raven and CRT scores as predictors (see column [5] in Table S2) whereas the effect of CRT becomes non-significant (*p* = 0.15). This result suggests that the significant effect of CRT scores on convergent thinking is driven more by cognitive ability (basic computational skills are also necessary for solving the CRT correctly) rather than by reflectiveness.

The relationship between our cognitive measures and divergent thinking is more complex. The models with the best fit report a linear and significant relationship between cognitive ability and all the measures of divergent thinking (all *p*'*s* < 0.03), except for flexibility (*p* = 0.22; see Table 2). Subjects with a higher Raven score tend to generate less uses (lower *fluency*), although these are more elaborated and original. Again, for these three creativity measures, one *SD* increase in Raven produces a variation in the dependent variable of about 20% of one *SD*. The effect of Raven on *flexibility* appears to be slightly U-shaped in Figure 1 but the regressions do not report any significant linear or quadratic relationship (all *p*'s > 0.22; see columns [1] and [2] in Table S5). As shown in columns [5] and [6] of Tables S3–S6, the effect of Raven on the divergent thinking measures remains virtually identical when controlling for CRT, which indicates that cognitive reflection does not mediate any of these relationships.

Contrary to the results observed with Raven, we do not find any significant linear relationship between cognitive styles and divergent thinking (all *p*'s > 0.28; see column [3] in Tables S3–S6). These results hold when we control for Raven (all *p*'s > 0.63; see column [5] in Tables S3–S6). However, we find a significant inverted U-shape relationship of CRT with both *fluency* and *flexibility*, as reported in Table 2 (*p* < 0.01 and *p* = 0.02, respectively). Subjects with an average level of cognitive reflection tend to produce more answers and use more categories than those subjects characterized by either a more intuitive or a more reflective cognitive style. Moreover, the fact that the coefficient of the linear term in the quadratic regression specification is not significantly different from zero in either case (*p* = 0.52 and *p* = 0.88, respectively) indicates that the maximum levels of fluency and flexibility are observed at the mean CRT score, as suggested by Figure 1. Effect sizes are comparable to those reported above insofar as, in both cases, moving one *SD* either above or below the mean CRT is associated with a decrease of about 20% of one *SD* in the dependent variable. Yet, the effects are larger for more extreme CRT values. Note that half of the observations fall outside the range mean ± one *SD* (see also Figure S1). Controlling for Raven does not alter these relationships (*p* = 0.01 and *p* = 0.02, respectively; see column [6] in Tables S4, S5), which again indicates an absence of mediation effects.

## Discussion

The dual-process approach of cognition has been recently suggested to reconcile previous conflictive findings on the relationship between creativity and executive cognition (Allen and Thomas, 2011; Ball et al., 2015; Barr et al., 2015; Sowden et al., 2015). We contribute to this literature by differentiating between the algorithmic and reflective minds (Evans and Stanovich, 2013), and by analyzing their separate effects on convergent thinking and four different dimensions of divergent thinking. We partially replicate the results of Barr et al. (2015) by finding that individuals' ability to make remote associations correlates positively with cognitive ability and cognitive reflection. However, we find that this effect on convergent thinking is mainly driven by cognitive ability. Similarly to Barr et al. (2015), we also find that higher levels of cognitive ability are related with higher originality scores and lower fluency scores in divergent thinking. Unlike Barr et al. (2015), we also analyze non-linear effects and find an inverted U-shape relationship between cognitive reflection and our measures of flexibility and fluency on the divergent thinking task. These new results suggest that individuals who are highly deliberative may have a disadvantage in producing a large number of new and creative ideas.

Dual-process models of creativity suggest that both generative and evaluative processes interact during the creative process (Finke et al., 1992; Basadur, 1995; Howard-Jones, 2002; Gabora, 2005; Nijstad et al., 2010; Gabora and Ranjan, 2013). Although these models do not have a straightforward mapping onto dual-process models of cognition, the interaction between Type 1 and Type 2 cognitive processes may play a different role in different phases of the creative process. In this line, Sowden et al. (2015) call for future research “…*to investigate the extent to which creativity is determined by the ability to shift between Type 1 and Type 2 thinking processes as a function of the circumstances and the stage of the creative processes*” (p. 55). Our results suggest that cognitive reflection, that is the disposition to override automatic responses related to Type 1 processing and engage in Type 2 controlled thought, has a complex effect on divergent thinking. To some extent, cognitive reflection may be necessary to shift between the generative and evaluative processes involved in the production of new ideas. However, individuals characterized by high levels of reflection may be less able to rely on their intuitive, autonomous mind which can also be needed for unleashing one's creative power (e.g., Dorfman et al., 1996; Norris and Epstein, 2011; Jarosz et al., 2012).

The finding of an inverted U-shape relationship between cognitive reflection (and, analogously, intuitive processing) and creativity is consistent with recent advances on the “mad genius hypothesis”: mild levels of top-down control dysfunction may be beneficial for creativity but severe impairment leads to poor creative performance (for a review, see Abraham, 2014).

Relatedly, neuropsychological research has shown an inverted-U shape relationship between spontaneous eye blink rates and *flexibility* in divergent creative thinking tasks (Chermahini and Hommel, 2010). To the extent that eye blink rates reflect dopaminergic activity (Karson, 1983), which is in turn linked to inhibitory control (Cohen and Servan-Schreiber, 1992), our results are in line with the finding of Chermahini and Hommel (2010).

Beyond its connection to basic cognitive research, our findings offer insights to managers in search for the creative talent of millennials. One essential implication of our study is that thinking too much may hamper important aspects of divergent creative thinking. This result is of primary relevance to hiring managers who may want to rely on cognitive reflection as the main criterion to recruit diligent (Corgnet et al., 2015b) and creative millennials. Our findings suggest that the cognitive tests used to recruit workers have to be adapted to the nature of the job offered. For example, recruiting for jobs that fundamentally require finding well-defined solutions to problems (such as accounting or actuarial jobs) can rely on a mix of cognitive ability and reflection tests which are good predictors of convergent creative thinking and diligence. However, recruiting for jobs that mainly require divergent creative thinking (such as marketing, industrial design, or psychology jobs) should not solely rely on cognitive measures. Recruiting based on cognitive reflection skills may actually prevent the hire of highly creative workers. These recommendations are becoming increasingly relevant as a growing number of jobs in modern economies require divergent creative thinking (Pink, 2005).

The current research has some necessary limitations that future research might remedy. To keep focus our study uses only one measure of fluid intelligence (Raven) and a single measure of cognitive style (CRT). Future research may assess the robustness of our findings to other measures of fluid intelligence and cognitive style, possibly extending the analysis to include crystallized intelligence. Also, our sample consisted entirely of undergraduates, with a limited age, education, and income range. Although this was a methodological choice that allowed us to study the workforce of the future, further studies may assess the robustness of our findings to different populations. Regarding our creativity measures, future research may attempt to extend our analysis to the case of practical creative tasks that are commonly encountered, for example, at the workplace. To that end, future research may embed the study of creativity in an organizational setting that allows for studying the relationship between workplace problem solving and cognitive skills.

On a methodological note, we used a fixed ordering of which may have influenced the results as, among other factors, fatigue may interfere with test results. While the 2-min break in the middle of the experiment might have mitigated spillover effects between the first and the second part of the experiment, concerns still remain. We encourage future research to explore possible ordering effects. In addition, future research focusing on state-level analyses of the role of intuition vs. reflection in creative performance is necessary to assess the robustness (and causality) of our trait-level findings as well as deepen our understanding of the cognitive basis of creativity. Along these lines, it would be interesting for future research to test the effect of cognitive manipulations such as cognitive load, ego depletion, priming, or time pressure/delay on creative performance. Our findings suggest that future research on the topic should attempt to capture potentially non-linear effects thus elaborating experimental designs that allow such effects to materialize. This can be done, for example, by considering at least three levels per treatment condition.

## Author contributions

All authors listed, have made substantial, direct and intellectual contribution to the work, and approved it for publication.

## Funding

The authors acknowledge financial support from the International Foundation for Research in Experimental Economics, the Argyros School of Business and Economics at Chapman University, the Spanish Ministry of Education [Grant 2012/00103/001], Ministry of Economy and Competence [2016/00122/001], Spanish Plan Nacional I+D MCI [ECO2013-44879-R], 2014-17, and Proyectos de Excelencia de la Junta Andalucía [P12.SEJ.1436], 2014-18.

### Conflict of interest statement

The authors declare that the research was conducted in the absence of any commercial or financial relationships that could be construed as a potential conflict of interest.

## Footnotes

^1^See the following press release: http://www.forbes.com/sites/susanadams/2012/09/24/older-workers-theres-hope-study-finds-employers-like-you-better-than-millennials/#1f5799cb4aa6 (accessed September 21, 2016).

^2^Positive effects of cognitive reflection on people's willingness to choose socially-efficient resource allocations (Lohse, 2016; Capraro et al., 2016) as well as to trust strangers (Corgnet et al., 2016) suggest other possible channels through which organizations may benefit from hiring individuals with a more reflective cognitive style. Cognitive reflection has also been found to play a key role in moral judgment (e.g., Paxton et al., 2012; Pennycook et al., 2014).
