# Supplementary material for: Creativity and Cognitive Skills among Millennials: Thinking Too Much and Creating Too Little
Source: Front Psychol. 2016 Oct 25;7:1626. doi: 10.3389/fpsyg.2016.01626 (PMC5078470; doi:10.3389/fpsyg.2016.01626)
Supplement: Supplementary file 1 [file Table1.PDF]

Table S1. Percentage of subjects answering correctly the CRT by question and gender.

CRT question	Males (%)	Females (%)	p-value
1	61.25	48.57	0.14
2	50.00	31.43	0.03
3	68.75	44.29	<0.01
4	55.00	31.43	<0.01
5	47.50	28.57	0.02
6	57.50	48.57	0.33
7	68.75	55.71	0.13

P-values from two-sided Fisher's exact tests for the (gender) difference in proportions.
