# Supplementary material for: Creativity and Cognitive Skills among Millennials: Thinking Too Much and Creating Too Little
Source: Front Psychol. 2016 Oct 25;7:1626. doi: 10.3389/fpsyg.2016.01626 (PMC5078470; doi:10.3389/fpsyg.2016.01626)
Supplement: Supplementary file 2 [file Table2.PDF]

TABLE S2. Linear and quadratic effect of cognitive abilities and cognitive styles on *RAT*.

	[1]	[2]	[3]	[4]	[5]	[6]
$\text{Raven}_{\text{std}}$	0.219***	0.238***			0.188**	0.198**
	(0.076)	(0.078)			(0.082)	(0.085)
$\text{Raven}^2_{\text{std}}$		0.047				0.023
		(0.061)				(0.060)
CRT_{std}			0.169**	0.164**	0.119	0.106
			(0.076)	(0.077)	(0.082)	(0.080)
$\text{CRT}^2_{\text{std}}$				0.059		0.055
				(0.081)		(0.083)
Constant	0.001	-0.050	-0.010	-0.072	-0.006	-0.089
	(0.080)	(0.102)	(0.080)	(0.121)	(0.079)	(0.149)
F	8.229	4.657	4.891	2.660	6.133	3.299
prob>F	0.005	0.011	0.029	0.073	0.003	0.013
R^2	0.053	0.056	0.030	0.033	0.067	0.070
Ll	-208.277	-208.017	-210.018	-209.801	-207.157	-206.918
AIC	420.555	422.034	424.036	425.602	420.315	423.836

Notes: OLS estimates. N=150. All variables are standardized. Robust standard errors are shown in parentheses. *p<0.05, **p<0.01, ***p<0.001
