# Supplementary material for: Creativity and Cognitive Skills among Millennials: Thinking Too Much and Creating Too Little
Source: Front Psychol. 2016 Oct 25;7:1626. doi: 10.3389/fpsyg.2016.01626 (PMC5078470; doi:10.3389/fpsyg.2016.01626)
Supplement: Supplementary file 3 [file Table3.PDF]

TABLE S3. Linear and quadratic effect of cognitive abilities and cognitive styles on *AUT Originality*.

	[1]	[2]	[3]	[4]	[5]	[6]
$Raven_{std}$	0.195***	0.215***			0.185**	0.207**
	(0.071)	(0.078)			(0.071)	(0.081)
$Raven^2_{std}$		0.049				0.047
		(0.065)				(0.069)
CRT_{std}			0.089	0.084	0.039	0.018
			(0.082)	(0.082)	(0.081)	(0.088)
CRT^2_{std}				0.059		0.058
				(0.092)		(0.089)
Constant	0.001	-0.052	-0.005	-0.068	-0.001	-0.113
	(0.080)	(0.112)	(0.081)	(0.122)	(0.080)	(0.148)
F	7.562	3.851	1.179	0.760	3.770	2.063
prob>F	0.007	0.023	0.279	0.470	0.025	0.089
R^2	0.042	0.046	0.008	0.011	0.043	0.049
Ll	-209.129	-208.845	-211.706	-211.488	-209.008	-208.591
AIC	422.258	423.689	427.411	428.975	424.015	427.181

Notes: OLS estimates. N=150. All variables are standardized. Robust standard errors are shown in parentheses. *p<0.05, **p<0.01, ***p<0.001
