# Supplementary material for: Creativity and Cognitive Skills among Millennials: Thinking Too Much and Creating Too Little
Source: Front Psychol. 2016 Oct 25;7:1626. doi: 10.3389/fpsyg.2016.01626 (PMC5078470; doi:10.3389/fpsyg.2016.01626)
Supplement: Supplementary file 4 [file Table4.PDF]

TABLE S4. Linear and quadratic effect of cognitive abilities and cognitive styles on *AUT Fluency*.

	[1]	[2]	[3]	[4]	[5]	[6]
$\text{Raven}_{\text{std}}$	-0.162**	-0.137			-0.157**	-0.122
	(0.074)	(0.088)			(0.074)	(0.092)
$\text{Raven}^2_{\text{std}}$		0.061				0.060
		(0.067)				(0.070)
CRT_{std}			-0.060	-0.043	-0.018	-0.025
			(0.069)	(0.066)	(0.067)	(0.069)
$\text{CRT}^2_{\text{std}}$				-0.207***		-0.196**
				(0.076)		(0.078)
Constant	-0.001	-0.068	0.003	0.224	0.000	0.144
	(0.081)	(0.111)	(0.083)	(0.136)	(0.082)	(0.169)
F	4.732	3.969	0.746	4.255	2.350	4.507
prob>F	0.031	0.021	0.389	0.016	0.099	0.002
R^2	0.029	0.035	0.004	0.039	0.029	0.068
Ll	-210.139	-209.701	-212.050	-209.359	-210.114	-207.096
AIC	424.278	425.401	428.100	424.717	426.228	424.192

Notes: OLS estimates. N=150. All variables are standardized. Robust standard errors are shown in parentheses. *p<0.05, **p<0.01, ***p<0.001
