# Supplementary material for: Creativity and Cognitive Skills among Millennials: Thinking Too Much and Creating Too Little
Source: Front Psychol. 2016 Oct 25;7:1626. doi: 10.3389/fpsyg.2016.01626 (PMC5078470; doi:10.3389/fpsyg.2016.01626)
Supplement: Supplementary file 5 [file Table5.PDF]

TABLE S5. Linear and quadratic effect of cognitive abilities and cognitive styles on *AUT Flexibility*.

	[1]	[2]	[3]	[4]	[5]	[6]
$Raven_{std}$	-0.099	-0.067			-0.105	-0.065
	(0.081)	(0.089)			(0.082)	(0.093)
$Raven^2_{std}$		0.078				0.069
		(0.074)				(0.077)
CRT_{std}			-0.005	0.011	0.023	0.012
			(0.075)	(0.073)	(0.074)	(0.076)
CRT^2_{std}				-0.194**		-0.183**
				(0.079)		(0.080)
Constant	-0.001	-0.086	0.000	0.206	-0.002	0.119
	(0.082)	(0.108)	(0.082)	(0.127)	(0.082)	(0.157)
F	1.490	1.384	0.004	2.990	0.837	2.234
prob>F	0.224	0.254	0.952	0.053	0.435	0.068
R^2	0.011	0.020	0.000	0.031	0.011	0.047
Ll	-211.522	-210.815	-212.337	-210.000	-211.481	-208.694
AIC	427.044	427.631	428.675	426.000	428.961	427.389

Notes: OLS estimates. N=150. All variables are standardized. Robust standard errors are shown in parentheses. *p<0.05, **p<0.01, ***p<0.001
