# Supplementary material for: Creativity and Cognitive Skills among Millennials: Thinking Too Much and Creating Too Little
Source: Front Psychol. 2016 Oct 25;7:1626. doi: 10.3389/fpsyg.2016.01626 (PMC5078470; doi:10.3389/fpsyg.2016.01626)
Supplement: Supplementary file 6 [file Table6.PDF]

TABLE S6. Linear and quadratic effect of cognitive abilities and cognitive styles on *AUT Elaboration*.

	[1]	[2]	[3]	[4]	[5]	[6]
$Raven_{std}$	0.246***	0.234***			0.249***	0.236**
	(0.067)	(0.080)			(0.072)	(0.092)
$Raven^2_{std}$		-0.027				-0.027
		(0.052)				(0.061)
CRT_{std}			0.053	0.053	-0.013	-0.002
			(0.082)	(0.083)	(0.084)	(0.095)
CRT^2_{std}				-0.004		-0.016
				(0.079)		(0.077)
Constant	0.001	0.031	-0.003	0.001	0.002	0.048
	(0.079)	(0.100)	(0.082)	(0.112)	(0.081)	(0.134)
F	13.302	9.870	0.420	0.209	6.628	4.896
prob>F	0.000	0.000	0.518	0.812	0.002	0.001
R^2	0.067	0.068	0.003	0.003	0.067	0.068
Ll	-207.174	-207.083	-212.112	-212.111	-207.160	-207.066
AIC	418.348	420.166	428.225	430.223	420.320	424.132

Notes: OLS estimates. N=150. All variables are standardized. Robust standard errors are shown in parentheses. *p<0.05, **p<0.01, ***p<0.001
