# Supplementary material for: Creativity and Cognitive Skills among Millennials: Thinking Too Much and Creating Too Little
Source: Front Psychol. 2016 Oct 25;7:1626. doi: 10.3389/fpsyg.2016.01626 (PMC5078470; doi:10.3389/fpsyg.2016.01626)
Supplement: Supplementary file 7 [file Presentation1.PDF]

Text S1. Cognitive Reflection Test

Taken from Frederick (2005):

(1) A bat and a ball cost $1.10 in total. The bat costs a dollar more than the ball. How much does the ball cost? ____ cents

[Correct answer: 5 cents; intuitive answer: 10 cents]

(2) If it takes 5 machines 5 minutes to make 5 widgets, how long would it take 100 machines to make 100 widgets? ____ minutes

[Correct answer: 5 minutes; intuitive answer: 100 minutes]

(3) In a lake, there is a patch of lily pads. Every day, the patch doubles in size. If it takes 48 days for the patch to cover the entire lake, how long would it take for the patch to cover half of the lake? ____ days

[Correct answer: 47 days; intuitive answer: 24 days]

Taken form Toplack, West and Stanovich (2014):

(4) If John can drink one barrel of water in 6 days, and Mary can drink one barrel of water in 12 days, how long would it take them to drink one barrel of water together? _____ days

[correct answer: 4 days; intuitive answer: 9]

(5) Jerry received both the 15th highest and the 15th lowest mark in the class. How many students are in the class? ______ students

[correct answer: 29 students; intuitive answer: 30]

(6) A man buys a pig for $60, sells it for $70, buys it back for $80, and sells it finally for $90. How much has he made? _____ dollars

[correct answer: $20; intuitive answer: $10]

(7) Simon decided to invest $8,000 in the stock market one day early in 2008. Six months after he invested, on July 17, the stocks he had purchased were down 50%. Fortunately for Simon, from July 17 to October 17, the stocks he had purchased went up 75%. At this point, Simon has: a. broken even in the stock market, b. is ahead of where he began, c. has lost money

[correct answer: c; intuitive response: b]
