# Supplementary figures and images for: Creativity and Cognitive Skills among Millennials: Thinking Too Much and Creating Too Little
Source: Front Psychol. 2016 Oct 25;7:1626. doi: 10.3389/fpsyg.2016.01626 (PMC5078470; doi:10.3389/fpsyg.2016.01626)

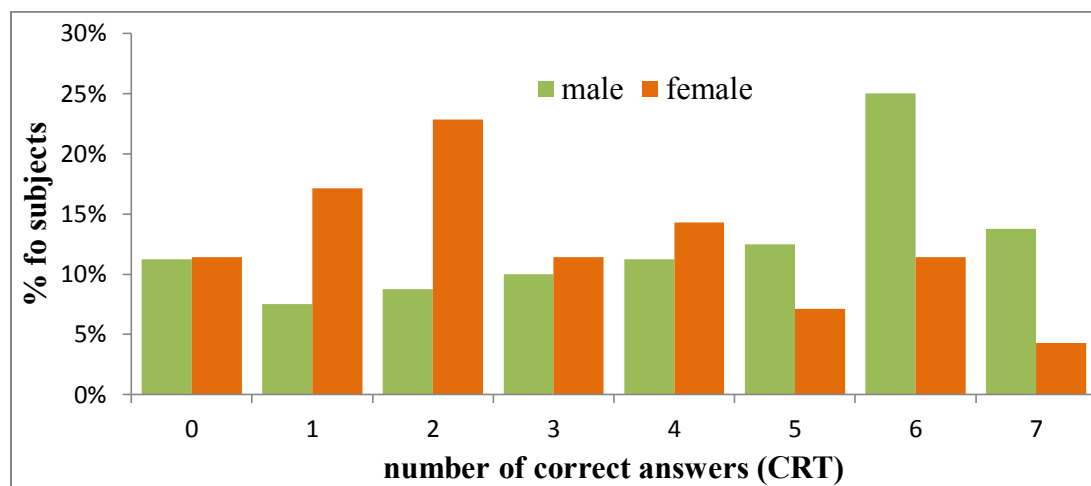

Figure S1. Distribution of CRT scores by gender

Supplement: Supplementary file 8 [file Image1.PDF]
